# A Design of Experiment Approach for Ionic Liquid-Based Extraction of Toxic Components-Minimized Essential Oil from *Myristica fragrans* Houtt. Fruits †

**DOI:** 10.3390/molecules23112817

**Published:** 2018-10-30

**Authors:** Daniela Lanari, Maria Carla Marcotullio, Andrea Neri

**Affiliations:** Dipartimento di Scienze Farmaceutiche, via del Liceo, 1- Università degli Studi, 06123 Perugia, Italy; daniela.lanari@unipg.it (D.L.); neriandrea1990@gmail.com (A.N.)

**Keywords:** *Myristica fragrans*, nutmeg, essential oil, ionic liquids, hydrodistillation, MODDE experimental design

## Abstract

The effect of the addition of ionic liquids (ILs) during the hydrodistillation of *Myristica fragrans* Houtt. (nutmeg) essential oil was studied. The essential oil of *M. fragrans* is characterized by the presence of terpenes, terpenoids, and of phenylpropanoids, such as methyl eugenol and safrole, that are regarded as genotoxic and carcinogenic. The aim of the work was to determine the best ionic liquid to improve the yield of the extraction of *M. fragrans* essential oil and decrease the extraction of toxic phenylpropanoids. Six ILs, namely 1,3-dimethylimidazolium chloride (**1**), 1,3-dimethylimidazolium dimethylphosphate (**2**), 1-(2-hydroxyethyl)-3-methylimidazolium chloride (**3**), 1-(2-hydroxyethyl)-3-methylimidazolium dimethylphosphate (**4**), 1-butyl-3-methylimidazolium chloride (**5**), and 1-butyl-3-methylimidazolium dimethylphosphate (**6**), were prepared by previously reported, innovative methods and then tested. An experimental design was used to optimize the extraction yield and to decrease the phenylpropanoids percentage using the synthesized ILs. The influence of the molarity of ILs was also studied. MODDE 12 software established 0.5 M 1-butyl-3-methylimidazolium chloride as the best co-solvent for the hydrodistillation of *M. fragrans* essential oil.

## 1. Introduction

*Myristica fragrans* Houtt. (Myristicaceae) is an evergreen tree native to the Moluccas, commonly known as the Spice Islands, and it is cultivated throughout Malaysia, India, Indonesia, and Southeast Asia [1]. The dried ovoid seed of this plant, covered by a red, ribbon-like, lacinated aril (mace) when fresh, is used for its aromatic properties in the culinary, pharmaceutical, and cosmetic industries [1]. *M. fragrans* (nutmeg) essential oil is used mainly to flavour soft drinks, canned foods, and meat products [1]. Furthermore, nutmeg is traditionally used in Ayurvedic medicine as an astringent, a carminative, and a sedative [2]. The flavour and the pharmacological properties [3] are mostly due to the presence of the essential oil (EO) that is traditionally prepared by hydrodistillation [4,5,6,7] and steam distillation [5,8], or by solvent extraction [9] through the use of innovative techniques [10]. More recently, SFE (supercritical fluid extraction) has been increasingly used as a technique to prepare EO [11,12,13].

Nutmeg EO is mostly constituted by monoterpenes (up to 90%) and phenylpropanoids. More than 30 components have been reported, with terpinen-4-ol, sabinene, and myristicin being the more abundant constituents of EO [13]. Phenylpropanoids such as eugenol, methyl eugenol, and methyl isoeugenol are also present in variable amounts, ranging from low to high percentages. For example, Morsy’s study found a total amount of alkenyl benzenes equal to 21.34% [14], while Du and coworkers found a total amount equal to 59.00% [4].

In the last several years, ionic liquids (ILs) have been considered a valid alternative to classic organic solvents as they feature relevant beneficial properties such as low vapor pressure, thermal and chemical stability, and non-flammability. ILs have been regarded as “green” additives in the extraction of EOs [15,16,17] due to their capability to dissolve lignocellulosic plant biomass and facilitate the extraction and separation of EO [18]. For this reason, their use as additives to improve essential oil and plant metabolite extraction yields has been considered [15,16,17,19,20,21,22,23].

Despite the advantages in replacing organic solvents with ILs, a toxicity profile for each class of ILs and the biodegradability issue should be taken into consideration to assess the environmental impact of the whole process [24,25].

In the present work, a variety of 1-alkyl-3-methyl imidazolium (MIM)-based ionic liquids have been tested to evaluate their influence in the hydrodistillation process.

The aims of the present study were (i) to evaluate the suitability of ILs as co-solvents for the extraction of *Myristica fragrans* EO, (ii) to study the effect of IL’s structure on the yield and composition of the EO extraction, and (iii) to optimize IL’s hydrodistillation using a design of experiment (DoE) approach.

## 2. Results and Discussion

Nutmeg EO shows different percentages of alkenyl benzenes. The Scientific Committee on Food (SCF) regarded safrole, methyleugenole, and estragole as toxic compounds due to their carcinogenic activity [26,27,28]; for this reason, any extraction condition that allows a diminished percentage of these compounds in the EO has to be regarded as valuable. On the other hand, there is evidence suggesting that many phenylpropenes are cytotoxic against several cancer cell lines [29], but most of the reports were not on clinical studies. These discrepancies could be ascribed to the formation of toxic metabolites in vivo [27].

**Figure 1:**
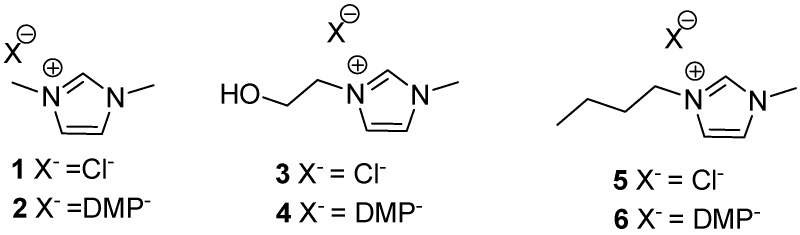
Tested 1-alkyl-3-methyl imidazolium (MIM)-based ionic liquids (ILs).

Among ILs, 1,3-dimethylimidazolium dimethylphosphate ([1,3-diMIM][DMP]) is well known to dissolve pulp [21,30], and we decided to test a variety of MIM-based ionic liquids with different substitution patterns in the imidazoyl moiety and with different counter ions, namely chloride (Cl^−^) and dimethylphosphate (DMP^−^) anions, as reported in Figure 1.

**Scheme 1:**
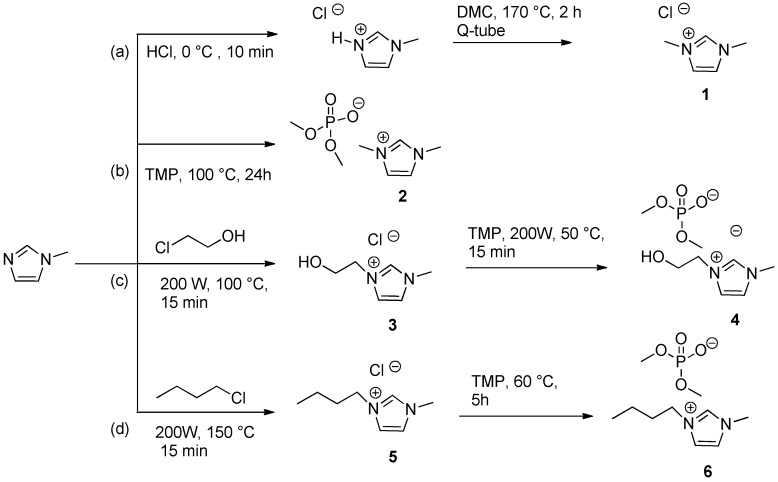
Preparation of ionic liquids (ILs). DMC: dimethyl carbonate, TMP: trimethylphosphate.

The synthesis of ILs **1**–**6** was performed either by using procedures used in previous literature or by modifying those protocols in order to obtain better results either in terms of yields or of sustainability. All ILs were synthesized starting from the common precursor 1-methylimidazole (MIM). 1,3-Dimethylimidazolium chloride **1** was prepared by a two-step procedure that first involved the synthesis of intermediate 1-methylimidazolium chloride after treatment of 1-methylimidazole with aqueous hydrochloric acid, followed by *N*-methylation with dimethyl carbonate (DMC) in a Q-tube apparatus (Scheme 1, path a). 1,3-Dimethylimidazolium dimethylphosphate **2** was synthesized by direct N-methylation of 1-methyimidazole with trimethylphosphate (TMP) by heating at 100 °C for 24 h (Scheme 1, path b). 1-(2-Hydroxyethyl)-3-methylimidazolium chloride **3** was synthesized from 1-methylimidazole and 2-chloroethanol under microwaves (MW) irradiation using an improved procedure from methods reported in literature, allowing us to employ almost stoichiometric ratios of reagents instead of an over-stoichiometric amount of the alkylating agent. This procedure resulted in excellent yield and an easier work-up. 1-(2-Hydroxyethyl)-3-methylimidazolium dimethylphosphate **4** was instead obtained by ion exchange of IL **3** in the presence of TMP under MW irradiation (Scheme 1, path c). 1-Butyl-3-methylimidazolium chloride **5** was obtained by the reaction of MIM with 4-chlorobutane under MW irradiation for 15 min, and 1-butyl-3-methylimidazolium dimethylphosphate **6** was synthesized through ion exchange with TMP under conventional heating (Scheme 1, path d).

To study the influence of ILs on the composition of the hydrodistilled EO, a traditional hydrodistillation of nutmeg was first performed using a 1:10 ratio of plant material and water and two, three, and four hours of distillation time. Using this procedure, we obtained an EO with a 0.86, 0.87, and 0.87% yield, respectively. As the yield of the extraction did not improve when the extraction time was extended, we decided to choose two hours as the extraction time. The essential oil obtained with these conditions was characterized by a high amount of phenylpropanoids (74.32%), of which 57.91% was constituted by myristicin and 2.90% by safrole. Since it has been described in literature that a presence of an inorganic salt in the extraction water could enhance the efficiency of the hydrodistillation process [31], we also used a 0.5 M solution of NaCl as reference electrolyte solution. The related yield was actually an intermediate value (0.98%) between that obtained using only water and those obtained using ILs. Neverthless, the composition of EO did not show a decrease in the percentage of phenylpropanoids (Table S1).

### Optimization of Hydrodistillation Conditions

The efficient extraction of plant metabolites from plant tissues is affected by several factors: the solvent, plant:solvent ratio, time of extraction, temperature, and the choice of the best extraction conditions, which can be a long and tedious process. Therefore, we felt the need for a design-based extraction to minimize the number of runs required to understand the factors affecting the process.

Using MODDE 12.0.1 software, we designed a set of experiments to determine which factor had a significant effect on the extraction yield of the EO and phenylpropanoids such as safrole.

The factors we examined were as follows: the nature of the anion (DMP^−^, Cl^−^), the nature of the cation (Figure 1), and the concentration of ILs in water (0.3 or 0.5 M). The measurements of the factors were optimized using a D-optimal design, resulting in 15 experiments plus three center points (Table 1).

After the synthesis of ILs, the experiments were carried out in random order to avoid systematic errors.

Table 1 shows the experimental design matrix and the corresponding response data for the yield of extraction and yield of phenylpropanoids in the EO (Tables S2–S8).

The model was fitted in MODDE 12.0 using partial least squares fitting (PLS).

**Figure 2:**
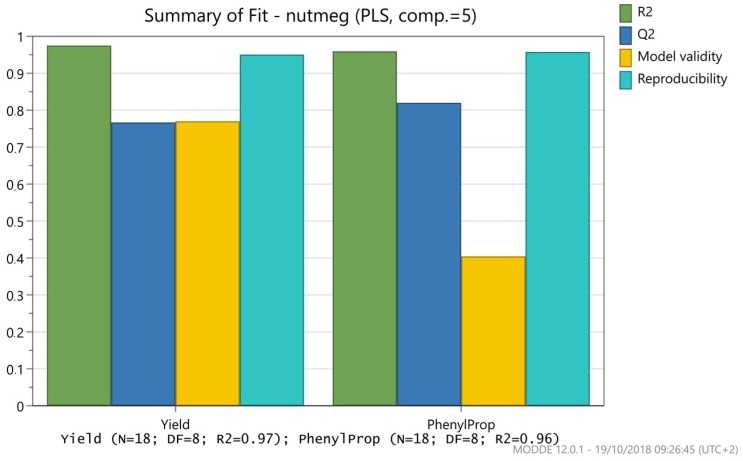
Summary of fit for the model of extraction yield and phenylpropanoids percentage. PLS: partial least squares fitting.

**Figure 3:**
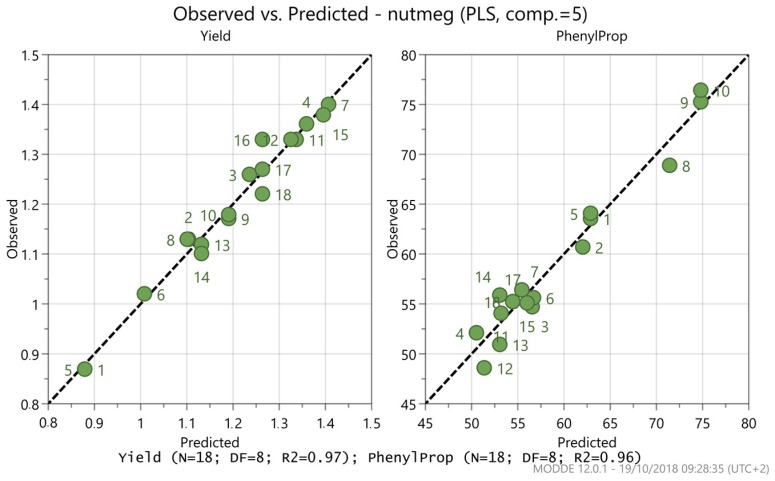
Plot of observed values versus predicted values for both responses.

The model showed good correlation coefficients (R^2^ = 0.97 and 0.96, respectively), good prediction values (Q^2^ = 0.72 and 0.82, respectively), and high reproducibility (0.95 and 0.87, respectively) (Figure 2). A plot of observed values versus predicted ones (Figure 3) showed that the model can be used to correlate variables and responses.

The ANOVA for the responses (yield of the extraction and percentage of phenylpropanoids in the EO) (Table 2) indicates that the model, with the *F*-values of 20.927 and 34.645 for phenylpropanoid percentage and yield, respectively, and a *p*-value of 0.000 for both, is significant.

**Figure 4:**
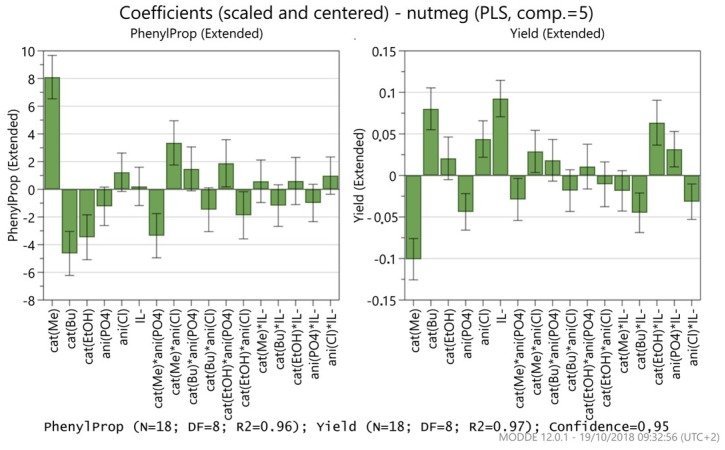
Plot of coefficient values for centered and scaled factors, which was obtained from partial least squares fitting (PLS) for the two response variables studied.

A summary of the effect of the factors on the responses is represented in Figure 4.

Looking to the *p*-values of each coefficient (Figure 4, Table S9), we can observe that seven factors have a significant effect on phenylpropanoid percentage (*p*-value < 0.05): nature of cation (all), quadratic term of cation [1,3-diMIM] × anion dimethylphosphate and [1,3-diMIM] × chloride, cation [1-EtOH-3-MIM] × anion dimethylphosphate, and [1-EtOH-3-MIM] × chloride. For the yield, eleven terms had significant results: cations [1,3-diMIM] and [1-But-3-MIM], both anions, ionic liquid molarity (IL), and quadratic terms [1,3-diMIM] × anion dimethylphosphate, [1,3-diMIM] × chloride, [1-But-3-MIM] × IL, [1-EtOH-3-MIM] × IL, dimethylphosphate × IL, and chloride × IL.

**Figure 5:**
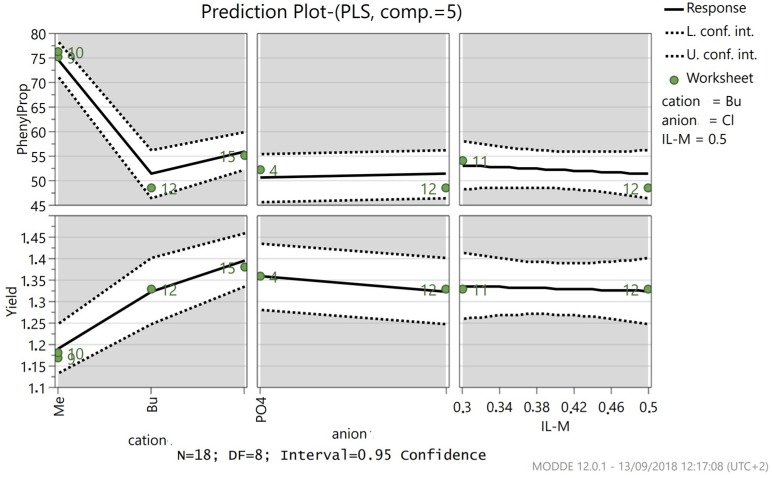
Prediction plot obtained by MODDE optimizer.

MODDE generated optimal conditions to increase the yield of extraction and to decrease the extraction of toxic phenylpropanoids that were identified in the use of 0.5 M of ionic liquid 1-butyl-3-methylimidazolium chloride **5** (Figure 5).

**Figure 6:**
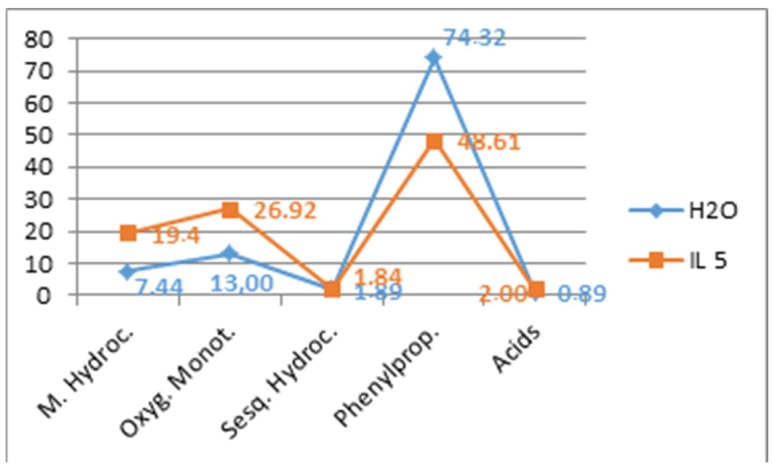
Comparison of essential oil composition extracted by water (blue) and by water–IL **5** (orange).

Figure 6 (Table S2) shows that the percentage of the volatile fraction (e.g., monoterpenes and oxygenated monoterpenes) is increased in the extraction aided by IL **5**, but a noteworthy finding was that the amount of toxic phenylpropanoids consistently dropped from a value of 74.32% to 48.61%.

A plausible explanation for the selective hydrodistillation of monoterpenes over phenylpropanoid components when a water–ILs mixture is used as a solvent could be found in the π–π stacking interaction between the MIM-based ionic liquids and the aromatic moiety of phenylpropanoids. It has been reported in literature [32] that ionic liquids with a 1,3-dimethyl-imidazolium cation could form clathrates in the presence of aromatic molecules; therefore, we can reasonably assume that an interaction occurs in solution between the ILs and the phenylpropanoid molecules that prevents, at least to some extent, their evaporation and recovery in the essential oil.

In order to further improve the new protocol for the extraction of nutmeg EO with a low content of phenylpropanoids, the possibility of recycling and reusing the IL was exploited. The water–IL **5** suspension used in a first extraction run was then collected after the removal of the organic matrix and reused three times. The yields and compositions of EO were analyzed for each run as reported in Table 3, and no noticeable change in yield or in the amount of phenylpropanoids was detected; therefore, recycling of the extraction mixture is possible for at least three times.

## 3. Materials and Methods

### 3.1. General Experimental Procedures

NMR spectra were recorded using a Bruker Avance DRX-400 and DPX-200 spectrometers (Bruker, Milan, Italy) operating at frequencies of 400 MHz (^1^H), 100 MHz (^13^C), 200 MHz (^1^H), and 50 MHz (^13^C). The spectra were measured in CDCl_3_ and DMSO-*d*_6_. The ^1^H- and ^13^C-NMR chemical shifts (δ) were expressed in ppm with reference to the solvent signals. Coupling constants are given in Hz. GC analyses were performed on a HP 6890N Network GC system (Hewlett Packard, Waldbronn, Germany) equipped with a Hewlett Packard MS 5975 mass selective detector (Hewlett Packard, Waldbronn, Germany) and a capillary column (DB-5MS; 30 m × 0.53 mm i.d., 0.50 μm film thickness, Agilent Technologies Italia, Cernusco sul Naviglio, Milan, Italy). The oven temperature was programmed from 40 °C for 7 min, then increased at 10 °C/min to 270 °C and held for 20 min. Injector and detector temperatures were 250 and 270 °C, respectively. Samples were dissolved in *n*-hexane to give 1 μL/mL solutions and were injected in the splitless mode using helium as carrier gas (1 mL/min); the injection volume was 1 μL. The ionization energy was 70 eV. Percentage compositions of the components were obtained from electronic integration dividing the area of each component by the total area of all components. The percentage values are the mean ± SD of two injections of the sample. All compounds were identified by the comparison of their retention indices (RI) relative to retention times on the DB-5MS column of a homologous series of C8–C20 alkanes with those reported in the literature [33] and by comparison of mass spectra from the Wiley 275 Mass Spectral Database (https://www.sisweb.com/software/ms/wiley.htm). Moreover, whenever possible, identification was confirmed by an injection of a standard commercial sample. All solvents were of analytical grade and were purchased from VWR (Milan, Italy) Deionized water was obtained by Milli-Q water purification system from Merck-Millipore (Milan, Italy). Ionic liquids were synthesized following literature procedures or with original protocols as reported in this manuscript.

### 3.2. Plant Material

Dried fruits of *M. fragrans* Houtt., purchased from Martin Bauer S.p.A. (Nichelino, TO, Italy) (lot A130016591/002), were generously provided by Aboca S.p.A. (Sansepolcro, Italy).

### 3.3. Hydrodistillation

Hydrodistillation was carried out for two hours using a water-recycling, Clevenger-type apparatus accordingly to the European Pharmacopoeia [34]. The essential oil produced was collected from the apparatus as a fragrant yellowish oil. The oil was dried over anhydrous Na_2_SO_4_ before being stored under an inert atmosphere at 4 °C prior to analysis. The reported yields are the mean of two distillations ± standard deviation (SD).

The coarsely chopped plant material (25 g) was suspended in a 500 mL spherical flask immediately before hydrodistillation with the following materials:

- 250 mL of deionized water,
- 250 mL of a 0.5 M NaCl solution,
- 250 mL of a 0.3 M solution of selected ILs in water,
- 250 mL of a 0.5 M solution of selected IL in water, and
- 250 mL of a 0.4 M solution of selected IL in water.

Residual water containing the dissolved IL of the best run was reused at least three times with fresh plant material without significant modification in oil composition and yield.

### 3.4. Design of Experiment

The MODDE v.12 software for Design of Experiments (DOE) and Optimization software (Sartorius Stedim, Malmo, Sweden) was used to optimise the extraction conditions. Three independent variables (factors), namely nature of the cation, nature of the anion, ratio ILs:water (molarity), at two levels and three replicates at the center point were studied. The two response variables studied were extraction yield and phenylpropanoids percentage in the oil. Eighteen experiments (N) were designed by the software. All the experiments were randomly performed with two replicates. To obtain the optimal conditions the model was fitted with PLS (Partial Least Squares) analysis.

### 3.5. Synthesis of Ionic Liquids

Synthesis of 1,3-dimethylimidazolium chloride (**1**) [35]. In a 250 mL round bottom flask immersed in an ice bath, 10 g of 1-methylimidazole was placed, and 10 mL of HCl (37% aqueous solution) was slowly added. After 20 min, the solvent was evaporated to yield the desired ionic liquid, 1-methylimidazolium chloride, to be used in the following step without further purification. In a Q-tube reactor, 3.0 g of 1-methylimidazolium chloride and 2.28 g of dimethyl carbonate (DMC; 0.025 mmol) were placed, and the mixture was heated at 170 °C for two hours to yield the desired product, 1,3-dimethyl imidazolium chloride **1**, in quantitative yield. ^1^H-NMR (200 MHz, DMSO-*d*_6_, ppm): δ = 9.46 (s, 1H), 7.87 (s, 2H), 3.87 (m, 6H).

Synthesis of 1,3-dimethylimidazolium dimethylphosphate (**2**) [19]. In a 250 mL round bottom flask, 21 mL of 1-methyl imidazole (0.263 mol) and 33.4 mL of trimethylphosphate (0.289 mol) were added. The mixture was stirred under heating (100 °C) for 24 h; then, it was washed with Et_2_O after cooling to remove residual TMP. The product 1,3-dimethylimidazoium dimethylphosphate was obtained in 95% yield (55.5 g). ^1^H-NMR (400 MHz, CDCl_3_, ppm): δ = 9.97 (s, 1H), 7.31 (s, 2H), 3.62 (s, 6H), 3.15 (d, 6H, *J* = 10.5 Hz).

Synthesis of 1-(2-hydroxyethyl)-3-methylimidazolium chloride (**3**) [36]. In an MW vial, 5 g of 1-methylimidazole (0.061 mol) and 5.39 g of 1-chloroethanol (0.067 mol) were consequently added, and then the reaction was irradiated (200 W) for 15 min at 100 °C. After cooling, the solid product that precipitated off the solution was washed with Et_2_O and then dried under vacuum to yield 8.89 g (0.05 mol) of 1-(2-hydroxyethyl)-3-methylimidazolium chloride 3 (90% yield). ^1^H-NMR (200 MHz, DMSO-*d*_6_, ppm): δ = 9.33 (s, 1H), 7.79 (m, 2H), 5.50 (bs, OH), 4.23 (t, 2H, 4.8 Hz), 3.86 (s, 3H), 3.67 (t, 2H, *J* = 4.8 Hz).

Synthesis of 1-(2-hydroxyethyl)-3-methylimidazoliumphosphate (**4**) [32]. In a MW vial, 0.286 g (1.76 mmol) of **3** and an equimolar amount of TMP (0.3 mL, 1.76 mmol) were placed, and the mixture was irradiated (200 W) for 15 min at 50 °C to yield the desired product 4 in quantitative yield. ^1^H-NMR (200 MHz, CDCl_3_, ppm): δ = 9.73 (s, 1H), 7.57 (m, 1H), 7.42 (m, 1H), 5.85 (bs, 1H), 4.39 (t, 2H, *J* = 4.7 Hz), 3.91 (s, 3H), 3.76 (t, 2H, J= 4.7 Hz), 3.53 (d, 6H, *J* = 11.3 Hz).

Synthesis of 1-butyl-3-methylimidazolium chloride (**5**) [37]. In a MW vial, 4 mL of 1-methylimidazole (0.0484 mol) and 7.6 mL of 1-chlorobutane (0.0726 mol) were consecutively added; then, the mixture was irradiated (200 W) at 150 °C for 25 min. Then, the supernatant 1-chlorobutane was removed and the mixture was then washed with hexane and diluted with CH_2_Cl_2_ to be transferred in a round bottom flask for the removal of solvent under vacuum. The desired product **5** was obtained in 90% yield. ^1^H-NMR (200 MHz, CDCl_3_, ppm): δ = 9.71 (s, 1H), 7.99 (m, 1H), 7.89 (m, 1H), 4.24 (t, 2H, *J* = 7.0 Hz), 3.91 (s, 3H), 1.77 (m, 2H), 1.30 (m, 2H), 0.88 (t, 3H, *J* = 7.2 Hz).

Synthesis of 1-butyl-3-methylimidazoliumphosphate (**6**) [38]. In a 250 mL round bottom flask, 20.25 g (0.11 mol) of **5** and 13.4 mL (0.11 mol) of TMP were consecutively added, and the mixture was heated at 60 °C for 5 h; the product 1-butyl-3-methylimidazolium dimethylphosphate was then obtained in quantitative yield. ^1^H-NMR (200 MHz, CDCl_3_, ppm): δ = 9.56 (s, 1H), 7.87 (m, 1H), 7.7 (m, 1H), 4.16 (t, 2H, *J* = 7.1 Hz), 3.83 (s, 3H), 3.30 (d, 6H, *J* = 10.6 Hz), 1.71 (m, 2H), 1.22 (m, 2H), 0.84 (t, 3H, *J* = 7.4 Hz).

## 4. Conclusions

In the present work, we studied the influence of different MIM-based ILs on the extraction yield and composition of *M. fragrans* EO. The optimum conditions for IL-assisted extraction were studied using a DoE (design of experiment) approach. Under the optimized conditions (extraction with water–IL **5** 0.5 M), an increase in the total yield (from 0.86% to 1.33%) was observed; furthermore, a reduction of 35% of the toxic phenylpropanoids was observed. Water–IL **5** extraction mixture was recovered and reused for three additional times, making this procedure even more convenient.

## Figures and Tables

**Table 1:** Factor level and design matrix for extraction yield and phenylpropanoids percentage.

Exp No.	Run Order	Cation ^a^	Anion ^b^	IL ^c^	IL-[M] ^d^	Yield % ^e^	Phenylpropanoids % ^f^
1	11	[1,3-diMIM]	DMP	**2**	0.3	0.87	63.57
2	18	[1,3-diMIM]	DMP	**2**	0.5	1.13	60.68
3	15	[1-But-3-MIM]	DMP	**6**	0.5	1.36	52.14
4	16	[1-But-3-MIM]	DMP	**6**	0.3	1.26	54.74
5	12	[1,3-diMIM]	DMP	**2**	0.3	0.87	64.07
6	3	[1-EtOH-3-MIM]	DMP	**4**	0.3	1.02	55.65
7	8	[1-EtOH-3-MIM]	DMP	**4**	0.5	1.40	56.38
8	7	[1,3-diMIM]	Cl	**1**	0.3	1.13	68.95
9	14	[1,3-diMIM]	Cl	**1**	0.5	1.17	75.23
10	5	[1,3-diMIM]	Cl	**1**	0.5	1.18	76.41
11	17	[1-But-3-MIM]	Cl	**5**	0.3	1.33	54.01
12	10	[1-But-3-MIM]	Cl	**5**	0.5	1.33	48.61
13	13	[1-EtOH-3-MIM]	Cl	**3**	0.3	1.12	50.90
14	9	[1-EtOH-3-MIM]	Cl	**3**	0.3	1.10	55.88
15	6	[1-EtOH-3-MIM]	Cl	**3**	0.5	1.38	55.16
16	4	[1-EtOH-3-MIM]	Cl	**3**	0.4	1.33	55.21
17	1	[1-EtOH-3-MIM]	Cl	**3**	0.4	1.27	55.18
18	2	[1-EtOH-3-MIM]	Cl	**3**	0.4	1.22	55.24

^a^ Cation: [1,3-diMIM]: 1,3-dimethylimidazolium, [1-But-3-MIM]: 1-butyl-3-methylimidazolium, [1-EtOH-3-MIM]: 1-(2-hydroxyethyl)-3-imidazolium; ^b^ Cl: chloride, DMP: dimethyl phosphate; ^c^ IL: see Figure 1; ^d^ IL-[M]: Molarity of ILs’ aqueous solutions; ^e^ Yields are the mean of two extractions; ^f^ Percentage obtained by FID peak-area normalization. Values are the mean of two analyses.

**Table 2:** ANOVA test for the model.

**Phenylpropanoids**	**DF**	**SS**	**MS (Variance)**	***F***	***p***	**SD**
Total corrected	17	1063.38	62.5519			7.90898
Regression	9	1020.05	113.339	20.9267	0.000	10.6461
Residual	8	43.3281	5.41601			2.32723
Lack of Fit (Model error)	3	30.1049	10.035	3.79445	0.093	3.1678
Pure error (Replicate error)	5	13.2232	2.64464			1.62623
**Yield**	**DF**	**SS**	**MS (Variance)**	***F***	***p***	**SD**
Total corrected	17	0.434362	0.0255507			0.159846
Regression	9	0.423497	0.0470552	34.6453	**0.000**	0.216922
Residual	8	0.0108656	0.0013582			0.0368538
Lack of Fit (Model error)	3	0.00454893	0.00151631	1.20025	**0.399**	0.0389398
Pure error (Replicate error)	5	0.00631667	0.00126333			0.0355434

DF: degrees of freedom, SS: sums of squares, MS: mean squares, *F*: *F*-value, *p*: *p*-value, SD: standard deviation.

**Table 3:** Recycling experiments using ionic liquid **5** 0.5 M.

Exp No	Yield %	Phenylpropanoids %
1	1.48	48.46
2	1.52	48.68
3	1.47	49.02
4	1.47	48.60

## Supplementary Materials

The supplementary materials are available online.
