# Supplementary material for: A Design of Experiment Approach for Ionic Liquid-Based Extraction of Toxic Components-Minimized Essential Oil from Myristica fragrans Houtt. Fruits
Source: Molecules. 2018 Oct 30;23(11):2817. doi: 10.3390/molecules23112817 (PMC6278261; doi:10.3390/molecules23112817)
Supplement: Supplementary file 1 [file molecules-23-02817-s001.pdf]

Supplementary Material

A Design of Experiment approach for Ionic Liquid-based Extraction of Toxic Components-Minimized Essential Oil from *Myristica fragrans* Houtt. Fruits.

Daniela Lanari[†], Andrea Neri and Maria Carla Marcotullio[‡,*]

Dipartimento di Scienze Farmaceutiche, via del Liceo, 1- Università degli Studi, 06123 Perugia, Italy; daniela.lanari@unipg.it; neriandrea1990@gmail.com; mariacarla.marcotullio@unipg.it

* Correspondence: mariacarla.marcotullio@unipg.it; Tel.: +39-075-5855100

[†]These authors contributed equally to this work

[‡]Dedicated to Professor M. Curini on the occasion of his 70th birthday

Summary

Notes to the Tables.. 1

Table S1. Composition of the essential oils of *Myristica fragrans* obtained by using NaCl 0.5M. 2

Table S2. Composition of the essential oils of *Myristica fragrans* obtained by common hydrodistillation and by using 0.5M IL **5**. 3

Table S3. Composition of the essential oils of *Myristica fragrans* obtained by using IL **1**....................... 5

Table S4. Composition of the essential oils of *Myristica fragrans* obtained by using IL **2**....................... 7

Table S5. Composition of the essential oils of *Myristica fragrans* obtained by using IL **3**....................... 8

Table S6. Composition of the essential oils of *Myristica fragrans* obtained by using IL **4**....................... 9

Table S7. Composition of the essential oils of *Myristica fragrans* obtained by using IL **5**..................... 10

Table S8. Composition of the essential oils of *Myristica fragrans* obtained by using IL **6**..................... 11

Table S9. *P*-values for all the coefficients.. 12

Notes to the Tables

[a] Percentage obtained by FID peak-area normalization. Values are presented as the mean of two extractions;

[b] Components are listed in order of their elution from a DB-5MS column;

[c] Identification: by comparison of the mass spectrum with those of the computer WILEY275 library (99% matching);

[d] NI: not identified.

Table S1. Composition of the essential oils of *Myristica fragrans* obtained by using NaCl 0.5M.

Component[a,b]	%
α-Thujene	0.20
α-Pinene	3.31
Sabinene	9.03
β-Pinene	5.08
Myrcene	0.72
α-Phellandrene	0.47
α-Terpinene	1.03
p-Cymene	0.96
Limonene	3.54
γ-Terpinene	1.94
cis-Sabinene hydrate	0.23
Terpinolene	0.62
Linalool	0.48
p-Menth-2-en-1-ol *cis*	0.36
p-Menth-2-en-1-ol *trans*	0.23
Terpinen-4-ol	8.25
α-Terpineol	0.95
trans-Piperitol	0.15
Bornyl acetate	0.13
Safrole	1.82
α-Cubebene	0.10
Eugenol	2.70
α-Copaene	0.57
Methyl eugenolo	3.27
β-Caryophyllene	0.20
Isoeugenol	0.44
Methyl isoeugenol	0.79
Myristicin	50.48
Elemicin	1.17
NI[d]	0.36
Myristic acid	
NI[d]	
**Total (%)**	**99.56**
**Total identified**	**99.21**
**Monoterpene hydrocar.**	**27.12**
**Oxygenated monoterpenes**	**10.55**
**Sesquiterpene hydrocar.**	**0.8/8**
**Phenols**	**60.66**
**Acids**	**0**

Table S2. Composition of the essential oils of *Myristica fragrans* obtained by common hydrodistillation and by using 0.5M IL **5**.

Component	H_2O (%)[a,b]	IL 5 (%)[a,b]	RI[c,d]	RI Lit.[e]
α-Thujene		0.11±0.10	925	930
α-Pinene	1.09±0.06	1.18±0.08	933	939
Sabinene	2.23±0.11	1.42±0.09	975	975
β-Pinene	1.62±0.08	1.65±012	978	979
Myrcene		0.37±0.07	989	990
α-Phellandrene	0.10±0.12	0.40±0.10	1004	1002
α-Terpinene	0.29±0.01	3.22±0.04	1015	1017
p-Cymene		1.04±0.012	1023	1024
Limonene	0.97±0.08	3.11±0.07	1030	1029
γ-Terpinene	0.57±0.07	5.01±0.05	1059	1059
cis-Sabinene hydrate	0.30±0.02	1.77±0.08	1070	1070
Terpinolene	0.27±0.05	0.14±0.07	1084	1088
Linalool	0.47±0.10		1099	1090
p-Menth-2-en-1-ol *cis*	0.44±0.11		1120	1121
p-Menth-2-en-1-ol *trans*	0.28±0.08		1140	1140
Terpinen-4-ol	10.03±0.01	19.57±0.14	1182	1177
α-Terpineol	1.45±0.16	4.91±0.12	1190	1188
trans-Piperitol	0.32±0.08		1214	1207
Bornyl acetate		2.44±0.08	1285	1288
Safrole	2.90±0.11	1.66±0.09	1289	1287
α-Cubebene	0.94±0.08	0.17±0.06	1344	1348
Eugenol	2.16±0.05	0.91±0.07	1356	1359
α-Copaene	0.62±0.10	0.30±0.03	1379	1376
Methyl eugenolo	7.15±0.16	3.51±0.12	1405	1403
β-Caryophyllene	0.33±0.15	1.37±0.08	1420	1417
Isoeugenol	0.24±0.14	0.20±0.09	1459	1447
Methyl isoeugenol	1.08±0.05	0.53±0.05	1504	1491
Myristicin	57.91±0.09	40.20±0.05	1522	1517
Elemicin	2.88±0.26	1.60±0.15	1556	1555
NI[f]	1.16±0.16	0.37±0.18	1647	
Myristic acid	0.89±0.40	2.00±0.25	1774	
NI[f]	0.87±0.21	0.55±0.09	1840	
**Total (%)**	**99.57**	**99.68**		
**Total identified**	**97.54**	**98.76**		
**Monoterpene hydrocarbons**	**7.44**	**19.40**		
**Oxygenated monoterpenes**	**13.00**	**26.92**		

**Sesquiterpene hydrocarbons**	**1.89**	**1.84**
**Phenols**	**74.32**	**48.61**
**Acids**	**0.89**	**2.00**

Table S3. Composition of the essential oils of *Myristica fragrans* obtained by using IL **1**.[a]

Component[b,c]	Exp. n° 8	Exp. n° 9	Exp. n° 10
α-Thujene	0.40	0.38	0.40
α-Pinene	4.17	2.55	2.08
Sabinene	10.65	8.32	7.61
β-Pinene	4.08	3.24	2.75
Myrcene	0.42	0.12	0.20
α-Phellandrene	0.13	0.29	0.20
α-Terpinene	0.15	0.12	0.10
p-Cymene	0.46	0.46	0.34
Limonene	0.48	0.58	0.43
γ-Terpinene	1.46	0.67	0.95
cis-Sabinene hydrate	0.39	0.38	0.38
Terpinolene	0.71	0.89	0.71
Linalool	0.87	0.26	0.60
p-Menth-2-en-1-ol *cis*	0.84	0.17	0.20
p-Menth-2-en-1-ol *trans*	0.13	0.11	0.14
Terpinen-4-ol	3.82	3.42	3.66
α-Terpineol	0.50	0.61	0.64
trans-Piperitol			
Bornyl acetate			
Safrole	2.31	2.09	2.50
α-Cubebene	0.13	0.15	0.12
Eugenol	0.80	0.62	0.63
α-Copaene	0.12	0.24	0.16
Methyl eugenolo	1.91	1.55	1.73
β-Caryophyllene	0.21	0.29	0.29
Isoeugenol	0.15	0.19	0.15
Methyl isoeugenol	0.20	0.35	0.15
Myristicin	62.80	69.29	70.50
Elemicin	0.78	1.14	0.75
NI[d]	0.14	0.16	0.15
Myristic acid	0.78	1.01	1.12
NI[d]			
**Total (%)**	**99.98**	**99.65**	**99.64**
**Total identified**	**99.84**	**98.49**	**98.49**
**Monoterpene hydrocar.**	**23.49**	**18.01**	**16.165**
**Oxygenated monoterpenes**	**6.16**	**4.56**	**5.24**
**Sesquiterpene hydrocar.**	**0.46**	**0.67**	**0.56**
**Phenols**	**68.95**	**75.23**	**76.41**
**Acids**	**0.78**	**1.01**	**1.12**

Table S4. Composition of the essential oils of *Myristica fragrans* obtained by using IL **2.**[a]

Component[b,c]	Exp. n° 1	Exp. n° 5	Exp. n° 2
α-Thujene	0.30	0.39	0.28
α-Pinene	2.40	2.72	3.26
Sabinene	7.11	7.03	
β-Pinene	2.31	2.14	7.88
Myrcene	0.56	0.67	3.42
α-Phellandrene			0.67
α-Terpinene	0.98	1.21	
p-Cymene	0.55	0.70	0.87
Limonene	1.82	1.97	0.94
γ-Terpinene	1.31	1.40	3.31
cis-Sabinene hydrate			2.45
Terpinolene	0.42	0.59	
Linalool	0.40	0.55	0.37
p-Menth-2-en-1-ol *cis*	0.31	0.48	0.31
p-Menth-2-en-1-ol *trans*			0.36
Terpinen-4-ol	8.09	8.23	0.39
α-Terpineol	1.72	1.69	9.59
trans-Piperitol	0.19	0.25	1.21
Bornyl acetate	0.14	0.15	0.19
Safrole	3.20	3.83	3.78
α-Cubebene	0.46	0.38	0.69
Eugenol	1.56	2.41	1.60
α-Copaene	0.56	0.51	0.64
Methyl eugenolo	3.54	5.86	4.45
β-Caryophyllene	0.56	0.57	0.28
Isoeugenol	0.31	0.45	0.22
Methyl isoeugenol	1.42	1.23	1.01
Myristicin	53.12	48.82	48.24
Elemicin	0.42	1.47	1.39
NI[d]	0.10	0.21	0.10
Myristic acid	3.14	3.76	1.88
NI[d]	0.42	0.16	
**Total (%)**	**97.42**	**99.81**	**99.76**
**Total identified**	**97.01**	**99.44**	**99.72**
**Monoterpene hydrocar.**	**17.77**	**18.81**	**23.44**
**Oxygenated monoterpenes**	**10.85**	**11.16**	**12.05**
**Sesquiterpene hydrocar.**	**1.58**	**1.46**	**1.61**
**Phenols**	**63.57**	**64.07**	**60.68**
**Acids**	**3.14**	**3.76**	**1.88**

Table S5. Composition of the essential oils of *Myristica fragrans* obtained by using IL **3**.[a]

Component[b,c]	Exp. n° 13	Exp. n° 14	Exp. n° 15
α-Thujene	1.07	0.98	0.82
α-Pinene	7.10	7.02	5.90
Sabinene	13.01	13.13	13.50
β-Pinene	1.80	2.86	3.22
Myrcene	1.16	0.62	0.78
α-Phellandrene	0.52	0.39	0.68
α-Terpinene	1.15	1.13	1.20
p-Cymene	0.90	0.69	0.92
Limonene	3.56	2.67	2.88
γ-Terpinene	1.50	1.78	1.94
cis-Sabinene hydrate	0.15	0.24	0.30
Terpinolene	0.71	0.47	0.39
Linalool	0.57	0.56	0.78
p-Menth-2-en-1-ol *cis*	0.43	0.30	0.56
p-Menth-2-en-1-ol *trans*	0.29	0.19	0.20
Terpinen-4-ol	9.90	7.33	7.28
α-Terpineol	1.34	0.99	1.03
trans-Piperitol	0.11	0.20	0.15
Bornyl acetate	0.10		0.28
Safrole	2.99	2.50	2.41
α-Cubebene	0.36	0.18	0.29
Eugenol	1.48	1.33	1.78
α-Copaene	0.32	0.26	0.33
Methyl eugenolo	4.82	3.90	3.50
β-Caryophyllene	0.14	0.12	0.20
Isoeugenol	1.05	0.94	0.77
Methyl isoeugenol	0.85	0.34	0.59
Myristicin	38.16	46.22	45.60
Elemicin	1.55	0.65	0.51
NI[d]	0.34	0.22	0.15
Myristic acid	2.21	1.43	0.29
NI[d]	0.13	0.32	
**Total (%)**	**99.81**	**99.96**	**99.21**
**Total identified**	**99.34**	**99.42**	**99.06**
**Monoterpene hydrocar.**	**32.63**	**31.98**	**32.51**
**Oxygenated monoterpenes**	**12.78**	**9.57**	**10.28**
**Sesquiterpene hydrocar.**	**0.82**	**0.56**	**0.82**
**Phenols**	**50.90**	**55.88**	**55.16**
**Acids**	**2.21**	**1.43**	**0.29**

Table S6. Composition of the essential oils of *Myristica fragrans* obtained by using IL **4.**[a]

**Component**[b,c]	**Exp. n° 6**	**Exp. n° 7**
α-Thujene	0.72	0.54
α-Pinene	5.18	3.76
Sabinene	7.63	2.10
β-Pinene	3.78	4.00
Myrcene	0.43	0.45
α-Phellandrene	0.19	0.59
α-Terpinene	0.20	2.38
p-Cymene	1.54	0.70
Limonene	2.59	2.68
γ-Terpinene	2.62	3.67
cis-Sabinene hydrate		
Terpinolene	0.56	0.83
Linalool	0.27	0.26
p-Menth-2-en-1-ol *cis*	1.25	0.12
p-Menth-2-en-1-ol *trans*	0.16	0.13
Terpinen-4-ol	12.27	14.39
α-Terpineol	1.20	1.15
trans-Piperitol	0.16	0.64
Bornyl acetate		
Safrole	1.55	2.51
α-Cubebene	0.15	0.15
Eugenol	0.90	1.36
α-Copaene	0.25	0.22
Methyl eugenolo	3.41	3.55
β-Caryophyllene		
Isoeugenol	0.42	0.34
Methyl isoeugenol	0.54	
Myristicin	47.38	46.51
Elemicin	1.47	2.11
NI[d]	0.13	0.43
Myristic acid	2.05	4.04
NI[d]	0.12	0.17
**Total (%)**	**99.09**	**99.82**
**Total identified**	**98.85**	**99.23**
**Monoterpene hydrocar.**	**25.44**	**21.70**
**Oxygenated monoterpenes**	**15.31**	**16.74**
**Sesquiterpene hydrocar.**	**0.40**	**0.46**
**Phenols**	**55.65**	**56.38**
**Acids**	**2.05**	**4.04**

Table S7. Composition of the essential oils of *Myristica fragrans* obtained by using IL **5.**[a]

Component[b,c]	Exp. n° 11	Exp. n° 12
α-Thujene	0.18	0.11
α-Pinene	1.35	1.18
Sabinene	2.33	1.42
β-Pinene	1.86	1.65
Myrcene	0.33	0.37
α-Phellandrene	0.30	0.40
α-Terpinene	2.03	3.22
p-Cymene	0.61	1.04
Limonene	2.43	3.11
γ-Terpinene	3.74	5.01
cis-Sabinene hydrate		1.77
Terpinolene	1.00	0.14
Linalool	0.24	
p-Menth-2-en-1-ol *cis*		
p-Menth-2-en-1-ol *trans*		
Terpinen-4-ol	19.76	19.57
α-Terpineol	2.47	4.91
trans-Piperitol	0..45	
Bornyl acetate	0.60	2.44
Safrole	2.70	1.66
α-Cubebene	0.19	0.17
Eugenol	1.30	0.91
α-Copaene	0.22	0.30
Methyl eugenolo	3.53	3.51
β-Caryophyllene	1.18	1.37
Isoeugenol	0.51	0.20
Methyl isoeugenol		0.53
Myristicin	44.11	40.20
Elemicin	1.85	1.60
NI[d]	0.33	0.37
Myristic acid	4.02	2.00
NI[d]		0.55
**Total (%)**	**99.59**	**99.68**
**Total identified**	**99.26**	**98.76**
**Monoterpene hydrocar.**	**16.13**	**19.40**
**Oxygenated monoterpenes**	**23.52**	**26.92**
**Sesquiterpene hydrocar.**	**1.58**	**1.84**
**Phenols**	**54.01**	**48.61**
**Acids**	**4.02**	**2.00**

Table S8. Composition of the essential oils of *Myristica fragrans* obtained by using IL **6.**[a]

Component[b,c]	Exp. n° 3	Exp. n° 4
α-Thujene		
α-Pinene	0.74	1.26
Sabinene	0.55	0.45
β-Pinene	1.26	1.55
Myrcene	0.19	0.30
α-Phellandrene	0.39	0.54
α-Terpinene	2.06	2.71
p-Cymene	0.51	0.56
Limonene	2.19	2.42
γ-Terpinene	3.99	4.59
cis-Sabinene hydrate		
Terpinolene	1.20	1.41
Linalool		0.20
p-Menth-2-en-1-ol *cis*		
p-Menth-2-en-1-ol *trans*		
Terpinen-4-ol	23.20	19.12
α-Terpineol	4.40	4.26
trans-Piperitol		
Bornyl acetate	2.11	2.04
Safrole	3.25	2.56
α-Cubebene	0.17	0.13
Eugenol	0.90	1.00
α-Copaene	0.25	0.17
Methyl eugenolo	2.81	3.20
β-Caryophyllene		1.53
Isoeugenol		0.14
Methyl isoeugenol	1.13	1.24
Myristicin	45.30	42.54
Elemicin	1.35	1.47
NI[d]		0.27
Myristic acid	1.59	2.67
NI[d]		
**Total (%)**	**99.94**	**98.31**
**Total identified**	**99.94**	**98.05**
**Monoterpene hydrocar.**	**13.07**	**15.78**
**Oxygenated monoterpenes**	**30.11**	**25.62**
**Sesquiterpene hydrocar.**	**0.42**	**1.83**
**Phenols**	**54.74**	**52.14**
**Acids**	**1.59**	**2.67**

Table S9. *P*-values for all the coefficients

PhenylProp	Coeff. SC	Std. Err.	P	Conf. int(±)
Constant	59,1907	0,572765	8,58677e-14	1,3208
cation	DF = 2			
cat(Me)	8,10179	0,680818	2,28486e-06	1,56997
cat(Bu)	-4,6353	0,689342	0,000148943	1,58962
cat(EtOH)	-3,46649	0,703149	0,00114951	1,62146
anion	DF = 1			
ani(PO4)	-1,23141	0,601773	0,0749363	1,38769
ani(Cl)	1,23141	0,601773	0,0749363	1,38769
IL-M	0,208447	0,599789	0,737156	1,38311
cation x anion	DF = 2			
cat(Me) x ani(PO4)	-3,35341	0,693404	0,00129444	1,59899
cat(Me) x ani(Cl)	3,35341	0,693404	0,00129444	1,59899
cat(Bu) x ani(PO4)	1,47396	0,687305	0,064319	1,58492
cat(Bu) x ani(Cl)	-1,47396	0,687305	0,064319	1,58492
cat(EtOH) x ani(PO4)	1,87945	0,738598	0,0344618	1,70321
cat(EtOH) x ani(Cl)	-1,87945	0,738598	0,0344618	1,70321
cat x IL-	DF = 2			
cat(Me)*IL-	0,579732	0,665761	0,409242	1,53524
cat(Bu)*IL-	-1,17732	0,651857	0,108537	1,50318
cat(EtOH)*IL-	0,597584	0,738941	0,44207	1,704
ani*IL-	DF = 1			
ani(PO4)*IL-	-0,98695	0,584587	0,129833	1,34806
ani(Cl)*IL-	0,98695	0,584587	0,129833	1,34806

Yield	Coeff. SC	Std. Err.	P	Conf. int(±)
Constant	1,20131	0,00907023	1,18091e-14	0,0209159
cation	DF = 2			
cat(Me)	-0,100847	0,0107813	1,39443e-05	0,0248618
cat(Bu)	0,0802443	0,0109163	7,98742e-05	0,0251731
cat(EtOH)	0,020603	0,011135	0,101431	0,0256772
ani	DF = 1			
ani(PO4)	-0,0438468	0,0095296	0,00175289	0,0219753
ani(Cl)	0,0438468	0,0095296	0,00175289	0,0219753
IL-M	0,0925136	0,00949818	1,03284e-05	0,0219028

**cat x ani**	DF = 2			
**cat(Me) x ani(PO4)**	-0,0289097	0,0109807	0,0300479	0,0253214
**cat(Me) x ani(Cl)**	0,0289097	0,0109807	0,0300479	0,0253214
**cat(Bu) x ani(PO4)**	0,0182545	0,0108841	0,132031	0,0250987
**cat(Bu) x ani(Cl)**	-0,0182545	0,0108841	0,132031	0,0250987
**cat(EtOH) x ani(PO4)**	0,0106553	0,0116963	0,388923	0,0269718
**cat(EtOH) x ani(Cl)**	-0,0106553	0,0116963	0,388923	0,0269718
**cat x IL-**	DF = 2			
**cat(Me) x IL-**	-0,0185567	0,0105429	0,116427	0,0243119
**cat(Bu) x IL-**	-0,0449436	0,0103227	0,00243267	0,0238042
**cat(EtOH) x IL-**	0,0635002	0,0117018	0,000625904	0,0269843
**ani x IL-**	DF = 1			
**ani(PO4) x IL-**	0,0316184	0,00925745	0,00914913	0,0213477
**ani(Cl) x IL-**	-0,0316184	0,00925745	0,00914913	0,0213477

1. Mary, L.; Rick, E.; María, T.; Ed, F., A retention index calculator simplifies identification of plant volatile organic compounds. *Phytochem. Anal* **2009,** 20, (5), 378-384.
2. Adams, R. P., *Identification of essential oil components by gas chromatography/mass spectrometry*. Allured Publishing Corporation: Carol Stream, 2007; p viii + 804 pp.
